# The habitat functional response links seasonal third‐order selection to second‐order landscape characteristics

**DOI:** 10.1002/ece3.5072

**Published:** 2019-03-28

**Authors:** Kelsey E. Paolini, Bronson K. Strickland, Jessica L. Tegt, Kurt C. VerCauteren, Garrett M. Street

**Affiliations:** ^1^ Department of Wildlife, Fisheries, and Aquaculture Mississippi State University Mississippi State Mississippi; ^2^ United States Department of Agriculture National Wildlife Research Center Fort Collins Colorado

**Keywords:** agroecology, autocorrelated kernel density estimator, habitat functional response, lasso, regularization, second‐order selection, shrinkage, third‐order selection

## Abstract

Determining how animals respond to differences in resource availabilities across spatiotemporal extents is critical to our understanding of organism distributions. Variations in resource distribution leading to changes in spatial arrangements across landscapes are indicative of a habitat functional response. Our goal was to assess how resource availabilities influenced both second‐order (i.e., home ranging behavior) and third‐order (i.e., habitat or resource selection) selection by feral pigs (*Sus scrofa*) in an agricultural landscape. We defined agriculturally based seasons to estimate home range characteristics using autocorrelated kernel density estimation within each season. We then modeled home range size as a function of resource availability (i.e., resource selection analyses) to determine whether individual behaviors were predicted by shifts in home ranging behavior. Both home range analyses and resource selection analyses indicated seasonal differences in selection for agricultural resources as availabilities changed, suggesting second‐ and third‐order selection is mechanistically linked through a habitat functional response.

## 1 INTRODUCTION

Animal home ranges represent the generalized areas traversed for daily activities and usage (Burt, 1943; Powell & Mitchell, 2012). The spatial location and arrangement patterns of animal home ranges are shaped by dynamic, integrated processes including dispersal (Bowman, Jaeger, & Fahrig, 2002), site fidelity (Powell, 2000), and landscape characteristics (Wiens, Stenseth, Horne, & Ims, 1993). The factors that generate home ranging behaviors lead to nonrandom use of the landscape because different resources are required throughout different life stages and across seasons (Börger, Dalziel, & Fryxell, 2008). As a result, organisms alter home range size, shape, and configuration in response to abiotic and biotic factors to increase their overall fitness (Börger et al., 2008; Wiens et al., 1993). Investigating specific mechanisms of home ranging behavior and incorporating appropriate temporal scales to study intraspecific variations in home ranging behaviors results in relevant biological inferences that can be generalized to broader landscapes (Börger et al., 2008, 2006).

A vast array of literature surrounds spatiotemporal influences on animal home ranges and emphasizes studying biologically relevant spatiotemporal extents to understand dynamic home range patterns (see Börger et al., 2008); however, a consistent theme of said literature is resource limitation (White, 1978). Resource availabilities fluctuate across spatiotemporal gradients whereby animals respond by changing their distribution across the landscape. These changes can occur across multiple spatial scales; for example, high‐quality forage availability had stronger negative effects on core home range size than on landscape‐level patterns of space use in moose (*Alces alces*) across temporal extents (van Beest, Rivrud, Loe, Milner, & Mysterud, 2011). Environmental characteristics strongly influence organism distributions and in some species such as the African buffalo (*Syncerus caffer*) more strongly determine home range size and shape relative to abiotic conditions (Naidoo et al., 2012). It is therefore paramount to study spatiotemporal changes in resource availability to understand how such environmental variables influence home ranging behaviors.

Dynamic home ranging behaviors are also influenced by habitat selection, which alters the distribution of organisms within their ranges (Marzluff, Millspaugh, Hurvitz, & Handcock, 2004). Habitat selection refers to the hierarchical decision‐making process whereby organisms select for the resources and conditions that meet biological requirements for species persistence based on innate and learned behaviors (Krausman, 1999). Hierarchical habitat selection (Johnson, 1980) depends on landscape‐scale selection which is often driven by selection at finer scales as a response to spatiotemporal dynamics of the surrounding environment (Beyer et al., 2010; Gaillard et al., 2010; Northrup, Anderson, Hooten, & Wittemyer, 2016). For example, woodland caribou (*Rangifer tarandus caribou*) respond to limiting factors most strongly at the largest spatial scale (i.e., seasonal geographic range), suggesting that animals situate their ranges within regions most conducive to survival (Rettie & Messier, 2000). Limiting factors regulate selection at successively coarser extents; if an animal requires a minimum amount of some limiting resource and occupies its landscape in such a fashion as to maximize access to that resource, we may expect to see substantial selection for the resource at finer scales as well. Yet animals respond at finer spatial scales to changes in forage availability by altering selection strength for different resources (i.e., habitat functional response; Mysterud & Ims, 1998), suggesting that the magnitude of selection is strongly context‐ and landscape‐specific.

Spatiotemporal dynamics in animal distributions indicate that home ranging behavior, habitat selection, and habitat functional responses are inherently linked through changes in environmental pressures and landscape composition. Consider two home ranging animals with identical resource requirements but that occupy landscapes of differing composition and net quality. Home range size and high‐quality resources are typically negatively correlated (van Beest, Mysterud, Loe, & Milner, 2010), meaning the animal in the lower quality landscape would by necessity expand its range to increase access to resources (2nd‐order selection; Johnson, 1980). As the range expands, the absolute availability of resources changes within the range. Modifications in seasonal home ranging behavior should evoke a change in the way the animal selects for habitat components within its range (3rd‐order selection) consistent with the habitat functional response (Johnson, 1980; Mysterud & Ims, 1998). This implies that seasonal home range characteristics such as size and composition are functionally related to habitat selection and use of space within the range.

**Figure 1:**
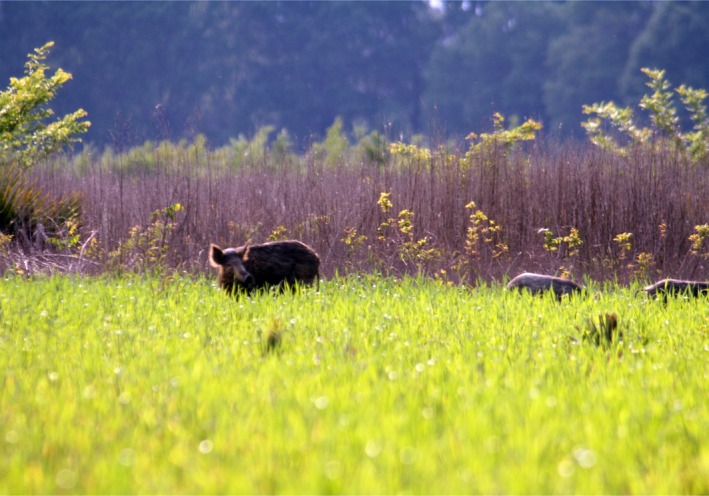
Feral pigs (*Sus scrofa*). Photo credit: Jay Cumbee

Here we investigate the interdependencies between seasonal home range characteristics and habitat selection in an invasive generalist, feral pigs (*Sus scrofa*; Figure 1). Feral pigs in the United States cause an estimated $1.5–2.5 billion in damages and control efforts within agricultural landscapes (D. Nolte, pers. comm.; Pimental, 2007). Therefore, a need exists to understand how feral pigs behaviorally respond within agricultural landscapes to uncover mechanisms influencing their space use and movements. Given a known strong preference for agricultural crops when available (Herrero, García‐Serrano, Couto, Ortuño, & García‐González, 2006; Schley & Roper, 2003), we expected feral pigs to change space utilization patterns to obtain increased access to critical resources as their availability changes within a range. This should evoke predictable changes in preference for habitat components. Specifically, we hypothesized that variation in feral pig home range size and composition (2nd‐order selection) is driven by seasonal changes in high‐quality resource availability (Börger et al., 2008; White, 1978), and that selection strength for resources within the range (3rd‐order selection) covaries with changing resource availability at the scale of the home range. We predicted that during the planting and growing seasons when food resources are readily abundant: (a) feral pig home range size will be negatively correlated with the relative abundance of corn (*Zea mays*) as a forage resource. During the harvest and fallow seasons, (b) home range size will be negatively correlated with wetland availability due to the absence of crop forage. We also predicted that (c) the selection strength of individuals for resources within the range will be correlated with changes in availability at the scale of the range itself. Lastly, (d) the correlation between resource selection and home range characteristics will be strongly season‐specific corresponding to behavioral and landscape changes.

## 2 METHODS

### 2.1 Study site

**Figure 2:**
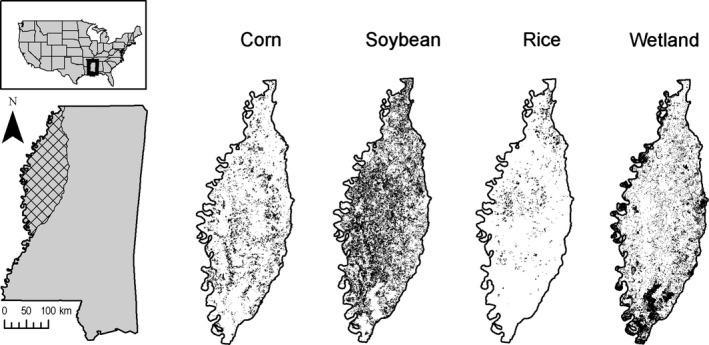
Location of study site within the northwestern region of the state of Mississippi, USA. The cross‐hatched area delineates the Mississippi Alluvial Valley. Submaps are rasters of availability of specific land‐cover types (dark areas represent greater availability)

This study occurred in northwestern Mississippi, known as the Lower Mississippi Alluvial Valley (LMAV; Figure 2). As a humid subtropical environment, the LMAV receives 145 cm of precipitation annually (Reinecke, Kaminski, Moorhead, Hodges, & Nassar, 1989). The site was a mosaic of bottomland hardwoods, including willow oak (*Quercus phellos*), water oak (*Quercus nigra*), and green ash (*Fraxinus pennsylvanica*), fragmented by agricultural fields which commonly include crops such as corn, soybean (*Glycine max*), and rice (*Oryza sativa*) fields (Reinecke et al., 1989; Stanturf, Schoenholtz, Schweitzer, & Shepard, 2001).

### 2.2 Data collection and management

During November 2015–March 2016, we opportunistically affixed 16 adult feral pigs with global positioning (GPS) collars (LOTEK Engineering Ltd., Newmarket, Canada; Vectronic Aerospace GmbH Berlin, Germany). Capturing and collaring procedures followed Mississippi State University's International Animal Care and Use Committee Protocol #14‐100, and the guidelines of the American Society of Mammalogists (Sikes & Gannon, 2011). Due to the nature of opportunistic trapping on locations with landowner approval, the possibility of collaring multiple individuals within the same sounder occurred. We calculated Pearson's correlation coefficients for all pairs of individuals against both latitude and longitude coordinates within a season. If the correlation coefficients were |*r*| > 0.5 for both coordinates, we deemed them nonindependent samples and removed the individual with less information (i.e., smaller number of recorded locations) from analyses. From all captured individuals (range of fixes per individual: 1,269–3,417; average fixes per individual: 2,312), our analyses indicated three individuals were dependent, and two were removed from the data set.

### 2.3 Seasonality

Our aim was to assess how agricultural phenology influences feral pig spatial utilization of temporally dynamic resources, therefore we developed temporal extents relevant to the different stages of agricultural phenology. Our temporal extents followed the general agricultural practices and phenology of corn within the region due to early planting (Sacks, Deryng, Foley, & Ramankutty, 2010) and its importance to the LMAV. We defined four distinct seasons: early growing (1 March 2016–15 May 2016); late growing (16 May 2016–31 July 2016); harvest (1 August 2016–31 October 2016); and fallow (1 November 2016–31 January 2017).

### 2.4 Resource selection analyses

We obtained agricultural landscape information for explanatory variables from the USDA National Agricultural Statistics Service Cropland Data Layer (CDL) with a 30 m × 30 m resolution (USDA NASS 2016). We compiled the agricultural delineations into corn, rice, soybean, wetland, other crop (i.e., were not a dominant crop type in the region), and other noncrop (i.e., mixed forest; see Paolini, Strickland, Tegt, Vercauteren, & Street, 2018 for a complete description of variable selection). We randomly sampled seasonal availability within each individual's 100% minimum convex polygon by creating a 1:1 used/available ratio. To assess relative resource selection, we generated 100 m buffers around each location and calculated percent cover by each crop type, respectively (Street et al., 2016). We conducted resource selection analyses using the *glmnet* package whereby we fit a generalized linear model with a logit link to each individual in a given season using the lasso to penalize maximum likelihood estimates and avoid model overfitting while simultaneously maximizing the predictive accuracy of the models (Friedman, Hastie, & Tibshirani, 2010).We averaged each individual model within a season to obtain season‐specific resource selection models.

### 2.5 Home range estimation

We measured variation in home range characteristics across seasons by calculating home range sizes for each individual within the respective seasons. We estimated 50% and 95% home ranges using the autocorrelated kernel density estimator (AKDE) using the *ctmm*package in R v. 3.4.0 (Calabrese, Fleming, & Gurarie, 2016; Fleming et al., 2015; R Core Team, 2017). We calculated an autocorrelation timescale for each individual because creating pooled population variograms does not accurately assess semivariance in animal locations over time when individuals have discontinuities in sampling schedules (Fleming & Calabrese, 2017).

### 2.6 Resource selection within the range

Resource selection analyses supported differences in seasonal preference (see Results). If 2nd‐order selection (i.e., home range placement as indicated by composition) changes as a function of relative availability of resources within the range, and if resource availability also influences habitat selection, then we expect the landscape characteristics of resource selection to influence home ranging behavior. We estimated the effects of the same resources that influenced 3rd‐order selection on seasonal home ranging behaviors. We created a single model per season for both 50% and 95% home range sizes using the *glmnet*package. In addition, we created partial residual plots to visualize the effect of each individual covariate on overall home range size.

## 3 RESULTS

**Figure 3:**
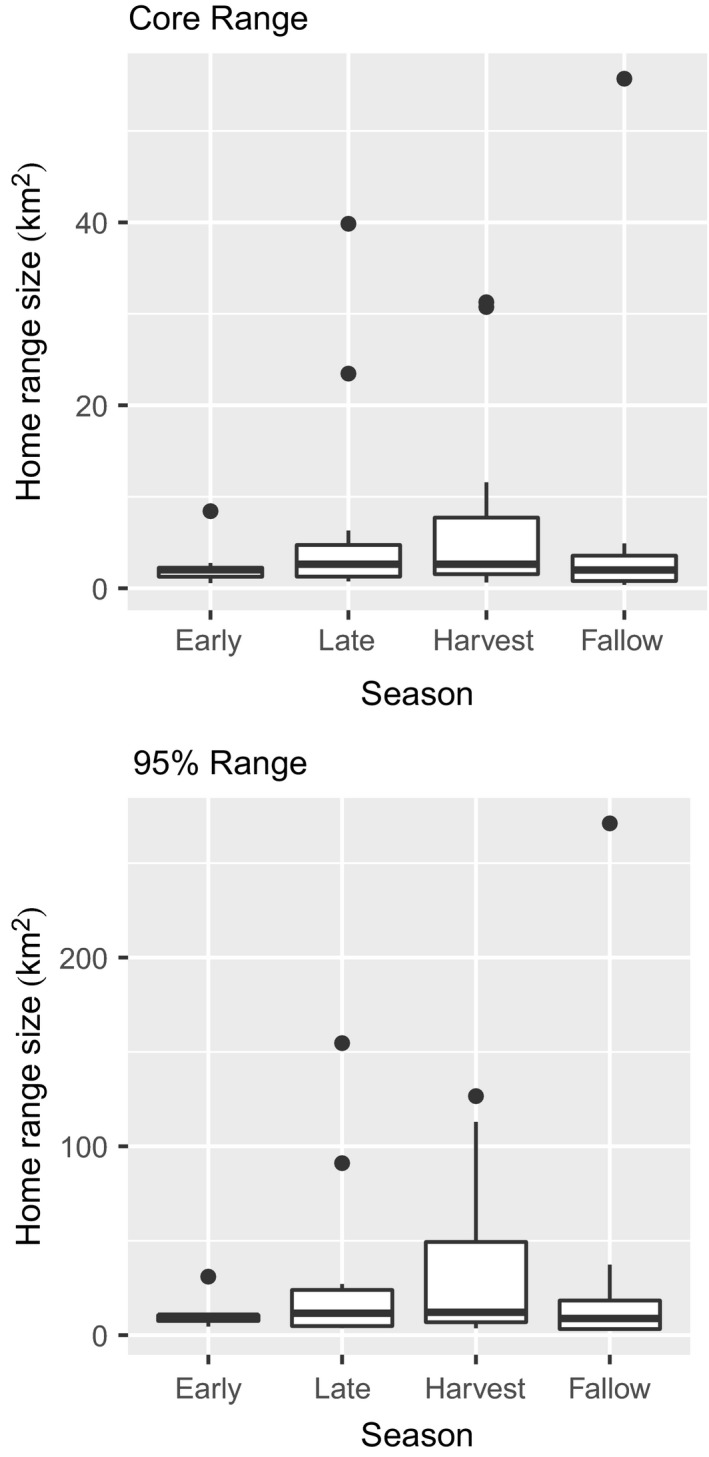
Box‐and‐whisker plots of home range size (km^2^) across agricultural seasons for the 50% and 95% home ranges derived from the autocorrelated kernel density estimator

Out of 16 feral pigs originally fitted with GPS collars, 13 provided viable data once nonindependent animals were removed. Individual animals were only included in home range analyses if relocation data spanned an entire season which included the following sample sizes: early growing (*n* = 8), late growing (*n* = 12), harvest (*n* = 11), and fallow (*n* = 8). We compared home range sizes across seasons using the Kruskal–Wallis test and found there were no significant differences in either the 50% or 95% ranges across seasons (*p* = 0.57 and 0.59, respectively). However, feral pigs showed considerably more variation in home range size within the harvest season for both 50% and 95% ranges (Figure 3).

### 3.1 Resource selection models

**Figure 4:**
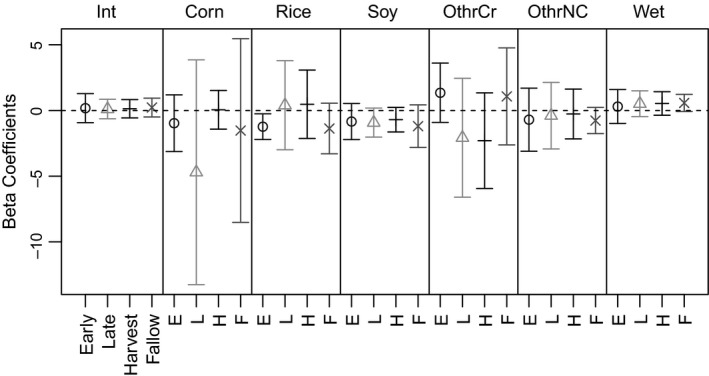
Beta coefficient plots for six landscape‐level predictors of 3rd‐order resource selection using the lasso within four agricultural seasons. Intervals are the empirical 95% intervals for beta coefficients estimated per animal

Seasonal availabilities of resources influenced selection by feral pigs. Both the direction and relative magnitude of selection varied for resources across seasons, with the exception of soybean, other noncrop, and wetland which only varied in relative magnitude (Figure 4). For the primary crops in the early growing season when grain availabilities increase, relative selection for corn, rice, and soybean was negative with corn having the largest variation in selection. During the late growing season, the relative magnitude of selection for corn and rice expanded, whereas the net selection for rice became positive. Resource selection patterns fluctuated during crop harvest whereby feral pigs became relatively neutral to corn and rice. Feral pigs selected for the other crop category in both the early growing season and fallow season when double cropped fields are planted and mature.

### 3.2 Partial residuals

**Figure 5:**
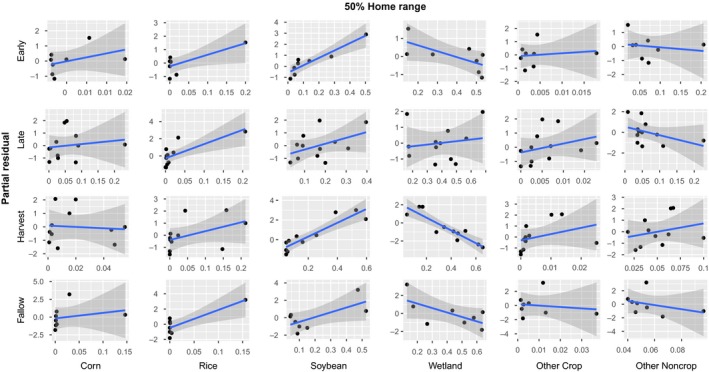
Partial residual plots for core (50%) home ranges for covariates across seasons estimated using the lasso

We calculated partial residual plots for home range models to visualize the relationships between model covariates and core home range size. Partial residual plots showed marked differences in the individual covariates as they influence overall home range sizes across seasons. Corn consistently had a positive relationship with 50% home ranges, with the exception of the harvest season where feral pigs became relatively neutral to corn (Figure 5). Both rice and soybeans had a positive relationship with 50% ranges throughout all seasons. The relationship with wetlands was negative in all seasons, apart from the late growing season where there was no influence, coinciding with large amounts of crop availabilities representing both food and cover. Other crops only influenced home ranges during the late in harvest season and feral pigs responded by expanding home ranges. No consistent patterns emerged with other noncrop and feral pig home ranges.

**Figure 6:**
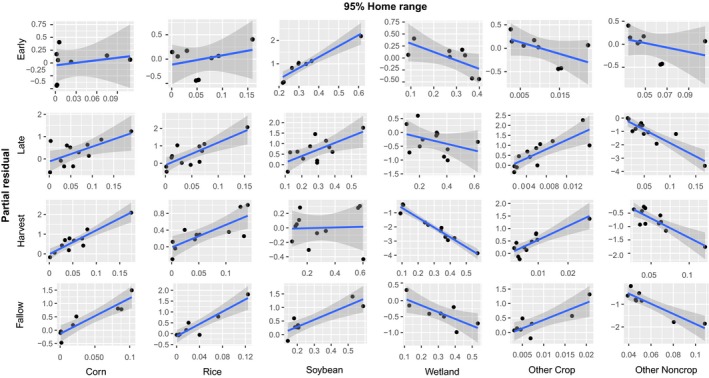
Partial residual plots for 95% home ranges for covariates across seasons estimated using the lasso

In addition, we found seasonal influences of covariates on 95% home ranges (Figure 6). Both corn and rice retained a positive relationship throughout all seasons. Similarly, a positive trend emerged for soybean except during the harvest season when its influence on home range size was relatively neutral. Wetlands consistently kept a negative relationship with 95% ranges, indicating an important resource for minimizing overall home ranging behavior. The other crop category only had a negative relationship in the fallow season where most crops no longer become available, while maintaining a positive relationship for the rest of the seasons. We found the same trend for other noncrop as with 50% home ranges having no distinct patterns across seasons. These seasonal variations indicate a predictable seasonal shift in the magnitude and direction of baseline resource selection driven by the relative availability of resources within the 50% and 95% home ranges—that is, a season‐specific habitat functional response (Mysterud & Ims, 1998).

## 4 DISCUSSION

The behavior of animals across spatial scales and levels of biological complexity is heavily dependent on landscape characteristics (Leblond et al., 2011; Northrup et al., 2016). Here we demonstrated that responses by home range size to landscape variables corresponding to forage availability differed across temporal extents (i.e., agricultural seasons) and often varied between spatial extents (i.e., 50% and 95% home ranges; Figures 5 and 6). The landscape characteristics influencing seasonal home range size at both the 50% and 95% isopleths also influenced baseline levels of resource selection by individuals; that is, the magnitude and direction of resource selection (i.e., 2nd‐order selection) changed with home range size via a season‐specific habitat functional response for characteristics of the home range itself (i.e., 3rd‐order selection; Figures 4, 5, 6; Johnson, 1980). In essence, 2nd‐ and 3rd‐order selection is mechanistically linked in a mutual feedback by the habitat functional response.

Effectively linking multi‐scale habitat selection to broader populations is a long‐standing topic in animal ecology. Multi‐scale habitat selection not only connects spatiotemporal extents to selection processes but also illustrates how organisms respond to their immediate environment (proximal causation) and how such behaviors subsequently affect organism fitness (ultimate causation; Hutto, 1985; Wiens, 1989; Mayor, Schneider, Schaefer, & Mahoney, 2009). Our study shows that temporal resource variation altered individual behaviors which led to changes in resource selection also influenced home range characteristics and ultimately indicated seasonal habitat functional responses (Figures 4, 5, 6). As landscape composition changes, organisms shift resource selection patterns to confer individual fitness. Consequently, home ranging behavior shifts to accommodate resource selection and as inferred here is linked to selection through the habitat functional response. Other studies have also stressed the importance of incorporating not only the relative availabilities of resources, but including home range sizes with availability to adequately detect habitat functional responses (Herfindal et al., 2009). Our approach informs how individuals respond behaviorally to their immediate environment by changing their distributions which can be used to infer limiting effects for organisms occupying a particular geographic region.

High‐quality resources generally reduce an organisms' distribution across a landscape because all physiological requirements can be met within a more concentrated area to increase individual fitness (van Beest et al., 2010). We expected the spatial utilization patterns of feral pigs to coincide with this trend as agricultural resources became more available; however, we did not find direct evidence supporting changes in overall home range sizes across seasons (Figure 3). We suspect this is due to dietary plasticity allowing feral pigs to efficiently sustain themselves throughout all seasons. This could also be attributed to the inherent correlation structure when using proportional land‐cover classification (i.e., as proportional cover by one category increases the rest decrease); however, we still found distinct seasonal trends at finer scales. Landscape characteristics in agroecosystems have been shown to be the primary driver of home ranging behavior, even in landscapes with intensive anthropogenic disturbances (Fattebert, Baubet, Slotow, & Fischer, 2017). In the LMAV, waste grain becomes readily abundant in the harvest season while additional crops from double planting practices (e.g., soybeans and winter wheat) and mast items are consumed in the fallow season (Reinecke et al., 1989; Schley & Roper, 2003). Although home range sizes were not influenced by changes in agricultural availability, we still found patterns of seasonal changes in landscape use (Figures 5 & 6). These results are consistent with seasonal habitat functional responses reliant upon the landscape composition (i.e., changes in agricultural availability).

Feral pig home range sizes were correlated with season‐specific covariates for both the core home range and broader space use (i.e., 50% and 95% AKDE, respectively). In agricultural landscapes, corn, rice, and soybean comprises a large amount of feral pig consumption when available (Herrero et al., 2006) and are primary crops planted within the LMAV. Core home ranges seasonally varied with season‐specific crops increasing with the relative abundance of crops (Figure 5), leading to changes in overall space use patterns with crop abundances across seasons. We suspect these changes occur due to different biological requirements within each season, meaning feral pigs are responding to the spatiotemporal environmental changes by altering their behaviors to exploit resources as the overall utility changes. For example, corn grain and kernels are important high‐quality food resources (Herrero et al., 2006) in the early growing and harvest season, whereas mature corn provides cover and thermoregulation in the late growing season. At broader extents, feral pigs change their behaviors by expanding their spatial distributions to increase the amount of relative crop availabilities of multiple types (e.g., corn, rice, and soybean, Figure 6). However, in the early growing season only soybean expanded home ranging behaviors and likely demonstrates changes in use over temporal extents. This concept is a fundamental component of habitat functional responses where both satiation and utilization of a resource through habitat selection play an integral part in determining species distributions (Mysterud & Ims, 1998).

Another important factor influencing feral pig spatiotemporal space use patterns was wetlands. Home range sizes generally decreased with increasing wetland availability at coarse extents (Figure 6), indicating the fundamental importance of wetlands to feral pig distributions across the LMAV. Feral pigs lack the physiological mechanisms to produce sweat (Mayer & Brisbin, 2009), meaning thermoregulatory facilitation is likely a strong behavioral driver (Street et al., 2016). An important factor to note was that core home ranges were most clearly influenced by wetlands in the harvest season when one would expect shelter provided by wetlands to be one of the main drivers of space use (Figure 6). This has two implications regarding feral pig behavior. First, it has been suggested that high human hunting pressures influence feral pig utilization of wetlands by driving movements to alternative cover sources (Gaston, Armstrong, Arjo, & Stribling, 2008). In the LMAV other large game species (i.e., white‐tailed deer, *Odocoileus virginianus*) are actively hunted which creates additional anthropogenic pressures influencing movements away from “ideal” wetland conditions. This implies the increased availability of high‐quality forage and shelter provided in agricultural fields, particularly when mast production is low in bottomland hardwoods, is an important driver in shifting core ranging behaviors. It is also important to note that because resource availabilities vary seasonally, it can appear as though they are no longer strongly selecting for that resource as it becomes readily available (i.e., that resource requirement is fulfilled and no longer need to select for the resource; Van Moorter, Visscher, Herfindal, Basille, & Mysterud, 2013). These patterns coincide with the dynamic distributions of other large ungulates where thermoregulatory and limiting resource availability drives changes in home range structure (van Beest et al., 2011; Dussault et al., 2005; Street et al., 2016).

The AKDE is a relatively new tool for home range estimation and uses autocorrelation in animal movement to better predict home ranges from use data (e.g., GPS relocations). Other studies have shown AKDE home ranges may produce biased estimates for some individuals which could be suggested here (Figure 4), but larger AKDE's are predicted when animal movements are minimal (Kay et al., 2017). We do not suspect this was the case due to the individuals' size, which was not included as a covariate to avoid model overfitting, and biological requirements to fulfill home ranging behavior. Additionally, even with relatively small sample sizes, we were still able to detect seasonal variations in animal home ranges corresponding to changes in agricultural availability. Because we were limited by small sample sizes, we emphasize using aggregate indicator variables (e.g., aggregations of crops) in place of multiple explicit predictors to assess broader patterns when data is limiting.

Our work stresses the importance of exploring multi‐scale habitat selection to characterize such spatiotemporal changes in animal space use patterns, as demonstrated here through integrating both home ranging behaviors and resource selection. Animal distributions across landscapes are driven by resource composition, distribution, and availability. For animals with site fidelity, this leads to changes in finer scale habitat selection patterns (e.g., within the home range). We found home ranging behavior by feral pigs in an agricultural landscape to be heavily influenced by changes in resource availability. These changes in resource availability, in turn, lead to changes in habitat selection acting through the habitat functional response. Understanding the relationships among landscape composition, habitat selection, and habitat functional responses provides a comprehensive assessment to study how organismal distributions vary across spatiotemporal scales. We suggest that future studies delve further into how other home range characteristics, such as measures of complexity (e.g., perimeter–area ratios, patch shape; Riitters et al., 1995), may shape habitat selection and utilization of the range to gain insight into the mechanisms producing restrictive space use patterns and how they relate to landscape composition and configuration.

## CONFLICT OF INTEREST

The authors declare that there is no conflict of interest.

## AUTHOR CONTRIBUTIONS

KEP, GMS, and BKS identified the research objectives; KEP performed data analysis with assistance from GMS; and GMS, BKS, JLT, and KCV acquired financial support for the project. All authors contributed to writing and editing.

## Data Availability

We have deposited our data at the public repository maintained by Mississippi State University (http://ir.library.msstate.edu/handle/11668/14341).

## References

[ece35072-bib-0001] Beyer, H. L. , Haydon, D. T. , Morales, J. M. , Frair, J. L. , Hebblewhite, M. , Mitchell, M. , & Matthiopoulos, J. (2010). The interpretation of habitat preference metrics under use‐availability designs. Philosophical Transactions of the Royal Society B: Biological Sciences, 365, 2245–2254. 10.1098/rstb.2010.0083 PMC2894962 20566501

[ece35072-bib-0002] Börger, L. , Dalziel, B. D. , & Fryxell, J. M. (2008). Are there general mechanisms of animal home range behaviour? A review and prospects for future research. Ecology Letters, 11, 637–650. 10.1111/j.1461-0248.2008.01182.x 18400017

[ece35072-bib-0003] Börger, L. , Franconi, N. , Ferretti, F. , Meschi, F. , De Michele, G. , Gantz, A. , & Coulson, T. (2006). An integrated approach to identify spatiotemporal and individual‐level determinants of animal home range size. The American Naturalist, 168, 471–485. 10.1086/507883 17004219

[ece35072-bib-0004] Bowman, J. , Jaeger, J. A. G. , & Fahrig, L. (2002). Dispersal distance of mammals is proportional to home range size. Ecology, 83, 2049–2055. 10.1890/0012-9658(2002)083[2049:DDOMIP]2.0.CO;2

[ece35072-bib-0005] Burt, W. H. (1943). Territoriality and home range concepts as applied to mammals. Journal of Mammalogy, 24, 346–352. 10.2307/1374834

[ece35072-bib-0006] Calabrese, J. M. , Fleming, C. H. , & Gurarie, E. (2016). ctmm: An R package for analyzing animal relocation data as a continuous‐time stochastic process. Methods in Ecology and Evolution, 7, 1124–1132.

[ece35072-bib-0007] Dussault, C. , Ouellet, J.‐P. , Courtois, R. , Huot, J. , Breton, L. , & Jolicoeur, H. (2005). Linking moose habitat selection to limiting factors. Ecography, 28, 619–628. 10.1111/j.2005.0906-7590.04263.x

[ece35072-bib-0008] Fattebert, J. , Baubet, E. , Slotow, R. , & Fischer, C. (2017). Landscape effects on wild boar home range size under contrasting harvest regimes in a human‐dominated agro‐ecosystem. European Journal of Wildlife Research, 63, 4683–4691. 10.1007/s10344-017-1090-9

[ece35072-bib-0009] Fleming, C. H. , & Calabrese, J. M. (2017). *ctmm*:*C* *ontinuous‐time movement modeling* . R package version 0.3.6.

[ece35072-bib-0010] Fleming, C. H. , Fagan, W. F. , Mueller, T. , Olson, K. A. , Leimgruber, P. , & Calabrese, J. M. (2015). Rigorous home range estimation with movement data: A new autocorrelated kernel density estimator. Ecology, 96, 1182–1188. 10.1890/14-2010.1 26236833

[ece35072-bib-0011] Friedman, J. , Hastie, T. , & Tibshirani, R. (2010). Regularization paths for generalized linear models via coordinate descent. Journal of Statistial Software, 33, 4683–22. 10.18637/jss.v033.i01 PMC2929880 20808728

[ece35072-bib-0012] Gaillard, J.M. , Hebblewhite, M. , Loison, A. , Fuller, M. , Powell, R. , Basille, M. , & Van Moorter, B. (2010). Habitat‐performance relationships: Finding the right metric at a given spatial scale. Philosophical Transactions of the Royal Society B: Biological Sciences, 365, 2255–2265. 10.1098/rstb.2010.0085 PMC2894964 20566502

[ece35072-bib-0013] Gaston, W. , Armstrong, J. , Arjo, W. , & Stribling, H. (2008). Home range and habitat use of feral hogs (Sus scrofa) on Lowndes County WMA, Alabama. National Conference on Feral Hogs, pp. 4683–18. St. Louis, MO.

[ece35072-bib-0014] Herfindal, I. , Tremblay, J. P. , Hansen, B. B. , Solberg, E. J. , Heim, M. , & Sæther, B. E. (2009). Scale dependency and functional response in moose habitat selection. Ecography, 32, 849–859. 10.1111/j.1600-0587.2009.05783.x

[ece35072-bib-0015] Herrero, J. , García‐Serrano, A. , Couto, S. , Ortuño, V. M. , & García‐González, R. (2006). Diet of wild boar *Sus scrofa* L. and crop damage in an intensive agroecosystem. European Journal of Wildlife Research, 52, 245–250. 10.1007/s10344-006-0045-3

[ece35072-bib-0016] Hutto, R. L. (1985). Habitat selection by nonbreeding, migratory land birds In Cody M. L. (Ed.), Habitat selection in birds (pp. 455–476). Orlando, FL: Academic Press Inc.

[ece35072-bib-0017] Johnson, D. H. (1980). The comparison of usage and availability measurements for evaluating resource preference. Ecology, 61, 65–71. 10.2307/1937156

[ece35072-bib-0018] Kay, S. L. , Fischer, J. W. , Monaghan, A. J. , Beasley, J. C. , Boughton, R. , Campbell, T. A. , … Pepin, K. M. (2017). Quantifying drivers of wild pig movement across multiple spatial and temporal scales. Movement Ecology, 5, 4683–15. 10.1186/s40462-017-0105-1 PMC5471724 28630712

[ece35072-bib-0019] Krausman, P. R. (1999). Some basic principles of habitat use. Grazing Behavior of Livestock and Wildlife, 70, 85–90.

[ece35072-bib-0020] Leblond, M. , Frair, J. , Fortin, D. , Dussault, C. , Ouellet, J. P. , & Courtois, R. (2011). Assessing the influence of resource covariates at multiple spatial scales: An application to forest‐dwelling caribou faced with intensive human activity. Landscape Ecology, 26, 1433–1446. 10.1007/s10980-011-9647-6

[ece35072-bib-0021] Marzluff, J. M. , Millspaugh, J. J. , Hurvitz, P. , & Handcock, M. S. (2004). Relating resources to a probabilistic measure of space use: Forest fragments and Steller's jays. Ecology, 85, 1411–1427. 10.1890/03-0114

[ece35072-bib-0022] Mayer, J. J. , & Brisbin, I. L. (2009). Wild pigs: Biology, damage, control techniques, and management. Aiken, SC: Savannah River National Laboratory.

[ece35072-bib-0023] Mayor, S. J. , Schneider, D. C. , Schaefer, J. A. , & Mahoney, S. P. (2009). Habitat selection at multiple scales. Ecoscience, 16, 238–247. 10.2980/16-2-3238

[ece35072-bib-0024] Mysterud, A. , & Ims, R. A. (1998). Functional responses in habitat use: Availability influences relative use in trade‐off situations. Ecology, 79, 1435–1441. 10.1890/0012-9658(1998)079[1435:FRIHUA]2.0.CO;2

[ece35072-bib-0025] Naidoo, R. , du Preez, P. , Stuart‐Hill, G. , Chris Weaver, L. , Jago, M. , & Wegmann, M. (2012). Factors affecting intraspecific variation in home range size of a large African herbivore. Landscape Ecology, 27, 1523–1534. 10.1007/s10980-012-9807-3

[ece35072-bib-0026] Northrup, J. M. , Anderson, C. R. , Hooten, M. B. , & Wittemyer, G. (2016). Movement reveals scale dependence in habitat selection of a large ungulate. Ecological Applications, 26, 2746–2757. 10.1002/eap.1403 27859842

[ece35072-bib-0027] Paolini, K. E. , Strickland, B. K. , Tegt, J. L. , Vercauteren, K. C. , & Street, G. M. (2018). Seasonal variation in preference dictates space use in an invasive generalist. PLoS ONE, 13, 4683–18. 10.1371/journal.pone.0199078 PMC6054371 30028855

[ece35072-bib-0028] Pimental, D. (2007). Environmental and economic costs of vertebrate species invasions into the United States. Managing Vertebrate Invasive Species: Proceedings of an International Symposium, 2–8.

[ece35072-bib-0029] Powell, R. A. (2000). Animal home ranges and territories and home range estimators In Boitani J., & Fuller T. K. (Eds.), Research techniques in animal ecology controversies and consequences (pp. 65–110). New York, NY: Columbia University Press.

[ece35072-bib-0030] Powell, R. A. , & Mitchell, M. S. (2012). What is a home range? Journal of Mammalogy, 93, 948–958.

[ece35072-bib-0031] R Core Team . (2017). R: A language and environment for statistical computing. Vienna, Austria: R Foundation for Statistical Computing Retrieved from https://www.R-project.org/

[ece35072-bib-0032] Reinecke, K. J. , Kaminski, R. M. , Moorhead, D. J. , Hodges, J. D. , & Nassar, J. R. (1989). Mississippi Alluvial Valley In Smith L. M., Pederson R. L., & Kaminski R. M. (Eds.), Habitat management for migrating and wintering waterfowl in North America (pp. 203–248). Lubbock, TX: Texas Tech University Press.

[ece35072-bib-0033] Rettie, W. J. , & Messier, F. (2000). Hierarchical habitat selection by woodland caribou: Its relationship to limiting factors. Ecography, 23, 466–478. 10.1111/j.1600-0587.2000.tb00303.x

[ece35072-bib-0034] Riitters, K. H. , O'Neill, R. V. , Hunsaker, C. T. , Wickham, J. D. , Yankee, D. H. , Timmins, S. P. , … Jackson, B. L. (1995). A factor analysis of landscape pattern and structure metrics. Landscape Ecology, 10, 23–39. 10.1007/BF00158551

[ece35072-bib-0035] Sacks, W. J. , Deryng, D. , Foley, J. A. , & Ramankutty, N. (2010). Crop planting dates: An analysis of global patterns. Global Ecology and Biogeography, 19, 607–620. 10.1111/j.1466-8238.2010.00551.x

[ece35072-bib-0036] Schley, L. , & Roper, T. J. (2003). Diet of wild boar *Sus scrofa* in Western Europe, with particular reference to consumption of agricultural crops. Mammal Review, 33, 43–56. 10.1046/j.1365-2907.2003.00010.x

[ece35072-bib-0037] Sikes, R. S. , & Gannon, W. L. ; The Animal Care and Use Committee of the American Society of Mammalogists . (2011). Guidelines of the American Society of Mammalogists for the use of wild mammals in research. Journal of Mammalogy, 92, 235–253. 10.1093/jmammal/gyw078 PMC5909806 29692469

[ece35072-bib-0038] Stanturf, J. A. , Schoenholtz, S. H. , Schweitzer, C. J. , & Shepard, J. P. (2001). Achieving restoration success: Myths in bottomland hardwood forests. Restoration Ecology, 9, 189–200. 10.1046/j.1526-100x.2001.009002189.x

[ece35072-bib-0039] Street, G. M. , Fieberg, J. , Rodgers, A. R. , Carstensen, M. , Moen, R. , Moore, S. A. , … Forester, J. D. (2016). Habitat functional response mitigates reduced foraging opportunity: Implications for animal fitness and space use. Landscape Ecology, 31, 1939–1953. 10.1007/s10980-016-0372-z

[ece35072-bib-0040] USDA National Agricultural Statistics Service Cropland Data Layer (2016). Published crop-specific data later [Online]. Retrieved from https://nassgeodata.gmu.edu/CropScape/

[ece35072-bib-0041] van Beest, F. M. , Mysterud, A. , Loe, L. E. , & Milner, J. M. (2010). Forage quantity, quality and depletion as scaledependent mechanisms driving habitat selection of a large browsing herbivore. Journal of Animal Ecology, 79, 910–922. 20443990 10.1111/j.1365-2656.2010.01701.x

[ece35072-bib-0042] van Beest, F. M. , Rivrud, I. M. , Loe, L. E. , Milner, J. M. , & Mysterud, A. (2011). What determines variation in home range size across spatiotemporal scales in a large browsing herbivore. Journal of Animal Ecology, 80, 771–785. 10.1111/j.1365-2656.2011.01829.x 21388373

[ece35072-bib-0043] Van Moorter, B. , Visscher, D. , Herfindal, I. , Basille, M. , & Mysterud, A. (2013). Inferring behavioural mechanisms in habitat selection studies getting the null‐hypothesis right for functional and familiarity responses. Ecography, 36, 323–330. 10.1111/j.1600-0587.2012.07291.x

[ece35072-bib-0044] White, T. C. R. (1978). The importance of a relative shortage of food in animal ecology. Oecologia, 33, 71–86. 10.1007/BF00376997 28309267

[ece35072-bib-0045] Wiens, J. A. (1989). Spatial scaling in ecology. Functional Ecology, 3, 385–397. 10.2307/2389612

[ece35072-bib-0046] Wiens, J. A. , Stenseth, N. C. , Van Horne, B. , & Ims, R. A. (1993). Ecological mechanisms and landscape ecology. Oikos, 66, 369–380. 10.2307/3544931

